# The cause of abdominal mass in a child with celiac disease: Rapunzel syndrome. A case report

**DOI:** 10.1590/1516-3180.2017.0281141017

**Published:** 2018-03-22

**Authors:** Yeliz Çağan Appak, Duygu Ertan, Miray Karakoyun, Gonca Özyurt, Tunç Özdemir, Maşallah Baran

**Affiliations:** I MD. Attending Physician, Department of Pediatric Gastroenterology, Tepecik Training and Research Hospital, Izmir, Turkey.; II MD. Pediatric Assistant, Department of Pediatrics, Tepecik Training and Research Hospital, İzmir, Turkey.; III MD. Attending Physician, Department of Pediatric Gastroenterology, Tepecik Training and Research Hospital, Izmir, Turkey.; IV MD. Assistant Professor, Department of Child and Adolescent Psychiatry, Tepecik Training and Research Hospital, Izmir Katip Celebi University School of Medicine, İzmir, Turkey.; V MD. Associate Professor, Department of Pediatric Surgery, Tepecik Training and Research Hospital, İzmir, Turkey.; VI MD. Associate Professor, Department of Pediatric Gastroenterology, Tepecik Training and Research Hospital, Izmir Katip Celebi University School of Medicine, İzmir, Turkey.

**Keywords:** Celiac disease, Abdomen, Bezoars, Trichotillomania, Depression

## Abstract

**CONTEXT:**

Rapunzel syndrome is a rare form of gastric trichobezoar that develops through outstretching of the bezoar from the stomach to the intestine.

**CASE REPORT:**

A 12-year-old girl who had been diagnosed with celiac disease six years earlier was brought to the department of pediatric gastroenterology because of abdominal distension. A palpable mass was detected. A trichobezoar that stretched to the small intestine was removed surgically. The patient was diagnosed as having anxiety and depressive disorder, and treatment started. Following the treatment, her previous trichophagia completely disappeared.

**CONCLUSION:**

Presence of trichobezoar should be kept in mind, especially when young girls who have psychiatric problems suffer from gastrointestinal symptoms.

## INTRODUCTION

Bezoars are associated with pica, mental retardation and psychiatric disorders among children.^1^ Hair pulling is defined as trichotillomania and eating hair is defined as trichophagia.^1^ The most frequently observed type of bezoar is trichobezoar, which develops in connection with hair deposition.^1^ The mass, which is generally composed of hair, accumulates among the mucosal folds of the stomach and expands over time. Because the rate of expansion is slow, symptoms only appear much later on, in most cases. Ninety percent of bezoars are found in adolescent girls.^1^

Rapunzel syndrome is a rare form of gastric trichobezoar and is formed by elongation of tail-like extensions from a bezoar, along the intestine. Trichophagia and trichotillomania may be observed together with depressive disorders, anxiety disorders and, particularly, obsessive-compulsive disorders.^2^

The case presented here involved presence of a trichobezoar with Rapunzel syndrome in a girl who was being followed up because of a diagnosis of celiac disease.

## CASE REPORT

The patient was a 12-year-old girl, who had been followed up for six years because of a diagnosis of celiac disease. She complained of a condition of painless abdominal distension.

The patient’s weight and height were in the normal range according to age. In the physical examination, a mass spreading from the left upper quadrant of the abdomen to the right upper quadrant, passing the middle line, was detected. Other systemic examinations were normal. The laboratory investigation of the case included the following results: white blood cells: 8,900/ul; hemoglobin: 12.4 mg/dl; hematocrit: 38.4%; thrombocytes: 337,000/ul; iron: 60 ug/dl; iron binding capacity: 250 ug/dl; folic acid: 11 ng/ml; ferritin: 15 ng/ml; and vitamin B12: 216 pg/ml. The results regarding tissue transglutaminase immunoglobulin A (Ig A) (27.58 RU/ml), anti-gliadin immunoglobulin A (IgA) and anti-endomysium antibody IgA were negative.

The patient was fully compliant with her gluten-free diet, according to her own declaration, and did not present any anemia. She showed significantly decreased tissue transglutaminase Ig A, which was > 200 RU/ml (normal level < 20 RU/ml) at the time when celiac disease was initially diagnosed. She was initially positive for anti-gliadin IgA and anti-endomysium IgA antibodies, at high titers, but she had become negative for these antibodies under her gluten-free diet recently.

**Figure 1:**
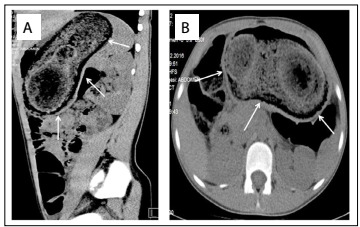
Computed tomography views: soft-tissue densities possibly compatible with a bezoar inside the stomach.

**Figure 2:**
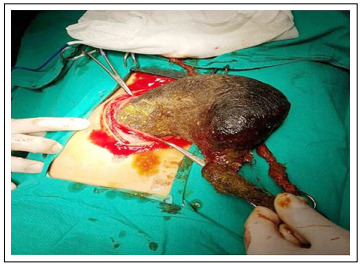
Surgical removal of the trichobezoar that filled the stomach and extended towards the small intestine.

There was no pathological finding from direct abdominal radiography. In the abdominal ultrasonography (USG), the radiologist could not detect any pathological condition. Through computed tomography (CT), most of the stomach could be seen and soft tissue densities that were possibly compatible with a bezoar were observed (Figure 1a, b). Surgical intervention was approved by the department of pediatric surgery. The trichobezoar, which had the shape of the stomach and stretched out towards the small intestine, was removed (Figure 2).

The patient had developed a habit of pulling out and eating her hair over the last year. She was reported to be successful at school, but she suffered from stress during examination periods and her relationships with friends was weak, which meant that she usually spent time on her own. Less hair was noticed in the frontal region of the skull. She underwent evaluation by a child psychiatrist, and this revealed that she was afraid of not being able to grow up adequately because of her celiac disease. Her communication with her peers was weak and she did not want to get involved in the social environment. She felt unable to calm herself down and she had initially started to pull out her hair and then started to eat it. The observation made at the end of her psychological assessment was that her anxiety was explicit and that she had depressive complaints. Administration of sertraline (50 mg/day) was started in connection with anxiety disorder and depressive disorder. During the follow-up at the department of child psychiatry, with cognitive and behavioral techniques therapy, the patient’s trichophagia regressed completely. The patient has been followed for the last year since the operation.

## DISCUSSION

Trichophagia and trichotillomania are particularly observed over the first two decades of life, and frequently in girls.^1^ The most frequent findings among children are epigastric mass, epigastric pain, nausea, vomiting, weight loss, diarrhea, constipation and hematemesis.^2^ The diagnosis may be delayed because of the non-specific symptoms. When the diagnosis is delayed, presence of trichobezoars may lead to serious complications. The most frequent complication is perforation of the stomach and small intestine. Invagination, pancreatitis, intestinal obstruction and peritonitis are infrequent complications.^1^^,^^2^

Direct radiography, abdominal USG and/or CT findings can be used in diagnosing trichobezoars.^3^ Upper gastrointestinal system endoscopy has the greatest sensitivity and specificity, given that it can provide information about the structure of the mass. The CT investigation can determine the existence, localization and distribution of the bezoars clearly.^3^ In our case, the bezoar could not be defined by means of USG, but could be viewed using CT.

Methods such as intragastric enzyme application (cellulose, pancreatic lipase, acetylcysteine, etc.), extracorporeal or endoscopic lithotripsy, break-up by means of laser and laparoscopic or open surgery can be used to treat bezoars. Endoscopic treatment is effective for phytobezoars and lactobezoars, since they are small in size and easily breakable, but it has less effect on trichobezoars. Nonetheless, it can be used for small-sized trichobezoars.^1^ Laparoscopic surgery is not advisable because of complications such as difficulty in breaking up the trichobezoar and the risk of obstruction of the intestines due to the broken pieces and deposition of hair in the abdominal cavity. The treatment advised for cases of large trichobezoars consists of the removal of the mass by means of laparotomy.^1^^,^^2^

Out of 10 papers in the literature regarding celiac disease, trichotillomania and bezoar (Table 1), only 6 were found to present reports on cases of these three pathological conditions occurring together (Table 2). Trichotillomania and trichophagia may be associated with neuropsychiatric disorders connected with celiac disease.^4^ Additionally, iron deficiency anemia and pica relating to celiac disease may be presented as the reason for presence of trichophagia and bezoars.^5^ Neuropsychiatric disorders and anemia secondary to celiac disease were ruled out in this patient’s case. Trichotillomania and trichophagia may also be observed together with serious chronic psychiatric disorders such as depressive disorders, anxiety disorders and, particularly, obsessive-compulsive disorders, and together with alcohol and drug addiction.^2^

**Table 1:** Articles relating to celiac disease and bezoar that were found through searching the medical literature databases (October 10, 2017)

Database	Search strategies	Papers found	Related papers
MEDLINE (via PubMed)	((celiac disease) OR coeliac disease) AND ((trichotillomania) OR bezoar)	1,061	10
	(“celiac disease”[Mesh]) AND “bezoars”[Mesh]	818	7
LILACS (via BVS)	(celiac disease)) AND (bezoar))	8	0

**Table 2:** Case reports on trichotillomania and trichobezoar with celiac disease reported in the literature

Author	Publication year	Diagnosis	Treatment
Larsson et al.^4^	2004	Trichobezoar was a result of celiac disease-induced pica.	Trichobezoar was removed via surgery; gluten-free diet was started.
Marcos Alonso et al.^5^	2005	Iron deficiency anemia and pica related to celiac disease.	Trichophagia regressed through gluten-free diet.
McCallum et al.^6^	2008	Iron deficiency anemia and pica related to celiac disease.	Trichobezoar retrieved through laparotomy; gluten-free diet was started.
Irastorza et al.^7^	2014	Neuropsychiatric disorder connected with celiac disease.	Trichophagia regressed through gluten-free diet.
Lihabi et al.^8^	2016	Trichotillomania was due to behavioral disorders secondary to celiac disease.	Trichotillomania improved through gluten-free diet.
Kalyoncu et al.^9^	2017	Trichotillomania was caused by behavioral disorders secondary to celiac disease.	Trichobezoar was removed through surgery; gluten-free diet continued.

The reason for trichophagia in our case was associated primarily with the depressive disorder and the anxiety disorder that were detected. Cognitive and behavioral techniques were applied during psychiatric consultations regarding this issue and antidepressant treatment was started. The patient’s trichophagia regressed completely through the treatments applied.

## CONCLUSION

Particularly when young girls with psychiatric problems complain of gastrointestinal system symptoms like palpable mass, abdominal pain and vomiting, they should be investigated for any history of trichophagia.

## References

[1] Eng K, Kay M (2012). Gastrointestinal bezoars: history and current treatment paradigms. Gastroenterol Hepatol (N Y).

[2] Frey AS, McKee M, King RA, Martin A (2005). Hair apparent: Rapunzel syndrome. Am J Psychiatry.

[3] Ripollés T, García-Aguayo J, Martínez MJ, Gil P (2001). Gastrointestinal bezoars: sonographic and CT characteristics. AJR Am J Roentgenol.

[4] Larsson LT, Nivenius K, Wettrell G (2004). Trichobezoar in a child with concomitant coeliac disease: a case report. Acta Paediatr.

[5] Marcos Alonso S, Bravo Mata M, Bautista Casasnova A, Pavón Belinchón P, Monasterio Corral L (2005). [Gastric trichobezoar as an atypical form of presentation of celiac disease]. An Pediatr (Barc).

[6] McCallum IJ, Van zanten C, Inam IZ (2008). Trichobezoar in a child with undiagnosed coeliac disease. J Paediatr Child Health.

[7] Irastorza I, Tutau C, Vitoria JC (2014). A trichobezoar in a child with undiagnosed celiac disease: a case report. World J Gastroenterol.

[8] Lihabi AA (2016). Trichotillomania in Celiac Disease. Case Rep Gastroenterol.

[9] Kalyoncu T, Çildir DA, Özbaran B (2017). Trichotillomania in celiac disease patient refractory to iron replacement. Int J Adolesc Med Health.

